# Magnitude of sugar-sweetened beverage consumption and associated factors among women aged 15–49 years old in two Sub-Saharan African countries

**DOI:** 10.1186/s12905-023-02814-1

**Published:** 2023-12-06

**Authors:** Birhan Ewunu Semagn, Abdulai Abubakari, Shimels Derso Kebede

**Affiliations:** 1 Department of Public Health, School of Public Health, Asrat Weldeyes Health Science Campus, Debre Berhan University, Debre Birhan, Ethiopia; 2 Department of Global and International Health, School of Public Health, University for Development Studies, Tamale, Ghana; 3 Department of Health Informatics, School of Public Health, College of Medicine and Health Science, Wollo University, Wollo, Ethiopia; 4 Department of Social and Behavioral Change, School of Public Health, University for Development Studies, Tamale, Ghana

**Keywords:** Sugar, Beverage, Unhealth diet, Non-communicable Disease, Africa

## Abstract

**Background:**

The consumption of Sugar-Sweetened Beverages (SSBs) has been linked to the global epidemic of obesity and chronic disease. Following the economic growth, urbanization, and attractive market for beverage companies, the consumption of SSBs is a rising public health challenge in low and middle-income countries. Hence, this study aimed to assess the magnitude of SSBs consumption and associated factors among women of reproductive age group in two SSA countries.

**Methods:**

This cross-sectional study used data from Integrated Public Use Micro Data Series-Performance Monitoring for Action (IPUMS-PMA) with a total sample of 3759 women aged 15–49 years old in Burkina Faso and Kenya. The data was collected on June - August 2018 in Burkina Faso, and May -August 2018 in Kenya. SSBs consumption was measured by asking a woman if she drank SSBs yesterday during the day or night, whether at home or anywhere else. A mixed-effect logistic regression model was employed to identify associated factors.

**Result:**

Half (50.38%) [95%CI; 46.04, 54.71] of women consumed SSBs. Sociodemographic characteristics like primary education (AOR = 1.35; 95%CI: 1.05–1.74), secondary education (AOR = 1.46; 95%CI: 1.13–1.90), being employed (AOR = 1.28; 95%CI: 1.05–1.56),and dietary characteristics like consumption of savory and fried snack (AOR = 1.61; 95%CI = 1.24–2.09), achieved minimum dietary diversity (AOR = 1.67; 95%CI: 1.38–2.01), moderate household food insecurity (AOR = 0.74, 95% CI: 0.58, 0.95), and sever household food insecurity (AOR = 0.71, 95% CI: 0.56, 0.89) had significant statistical association with SSBs consumption.

**Conclusion:**

Consumption of SSBs among women in two Sub-Saharan African countries (Burkina Faso and Kenya) is high. Having higher educational status, being employed, achieved minimum dietary diversity, and having low/no household food in-security were found to be significantly associated with SSBs compared with their counterparts. We recommend for further study in other African countries using objective measurements of SSBs consumption.

## Background

One of an ongoing public health problem around the world is the consumption of Sugar -Sweetened Beverages (SSBs) above the daily limits for free sugar [1, 2]. SSBs consumption includes the intake of all type of beverages containing free sugars like fruit juices, soft drinks/fizzy drinks, chocolate drinks (including those made with powders), sweet tea or coffee with sugar, fortified sweet drinks, malt drinks and energy drinks [3, 4]. Following the economic growth, urbanization and attractive market for beverage companies in low- and middle-income countries, the consumption of SSBs is now on the rise [1, 2].

The consumption of SSBs has been linked to the global epidemic of obesity and chronic disease [1]. Though it’s not an exclusive cause of non-communicable disease different studies have shown the association between higher consumption of SSBs with the risk of type2 diabetes mellitus, obesity, hypertension, cancer, heart disease, kidney disease, bone disease, and all-cause mortality [5–9]. Moreover, consumption of SSBs has been associated with less healthy behaviors like low physical exercise, low consumption of fruit/vegetables, and more screen time (television, cell phones, computers and video games) [10, 11]. Furthermore, SSBs consumption among women has been linked with increased risk of breast cancer mortality [12, 13], gestational hypertension [14], periodontal disease [15], early onset colorectal cancer [16], liver cancer [17], postpartum weight gain [18], diabetes [19], depression [20], stroke, coronary heart disease, and all-cause mortality [21]. Also, evidence highlights the greater health risk of sugary soft drinks compared to sugar-containing foods [22].

Previous research conducted among different population groups mainly in developed countries identified socio demographic characteristics (younger age, urban residence, household income), health characteristics (perceived overweight, obesity, diagnosis of heart disease or depression), and dietary characteristics (fruit consumption, having had processed meat, fried food from street vendors) as factors associated with SSBs consumption [23–25].

Though there is limited evidence on SSBs sale and consumption in Kenya, in 2018/19 a 30% increase in sugar production was forecasted with estimated annual consumption of 800,000 metric ton [26].

The increased consumption of SSBs in low- and middle-income countries, call for evidence based public health interventions to tackle the problems early [2, 7, 27]. Meanwhile there is dearth of knowledge on magnitude of SSBs consumption and associated determinants in SSA. This study aimed to (1) assess the magnitude of SSBs consumption among women of reproductive age group in two Sub-Saharan African countries and, (2) Identify factors that are associated with SSBs consumption among women of reproductive age group in two SSA countries This study may serve as a baseline to guide the development of targeted interventions to address diet related non-communicable diseases.

## Methods

### Study setting

This study is conducted in two Sub-Saharan Africa countries (Kenya and Burkina Faso). Kenya is a lower middle income country in East Africa with 47 administrative counties and population size of above 55 million [28]. About 34% of households in Kenya are headed by women. Also, about half (45%) of women in reproductive age (20–49) in Kenya are obese or overweight [29].

Burkina Faso is a low income landlocked Sahelian country in West Africa. The country is divided into 13 regions, 45 provinces and 351 municipalities [30]. The total population of Burkina Faso as at 2019 was about 21 million with 51.7% being women [31].

Global Nutrition Report estimates show that Burkina Faso has shown inadequate progress towards achieving the diet related non-communicable disease (NCD) targets as 10.1% of adult women and 3.4% of adult men are obese. In the same vein, diabetes is estimated to affect 6.6% of adult women and 9.3% of adult men [32].

### Study design, and data source

Cross sectional study design was used. The data was obtained from the Integrated Public Use Micro data Series-Performance Monitoring for Action (IPUMS-PMA) website [33]. The data was initially collected by a project called PMA 2020. PMA 2020 is a project that collects data on households, women, and service delivery points (i.e., health facilities) in 11 priority countries that have pledged to participate in the Family Planning 2020 (FP2020) effort. From 2017 to 2018, the PMA2020 program piloted a new nutrition survey module in two Sub-Saharan African Countries (Burkina Faso and Kenya). IPUMS PMA is another project that provides an interactive web dissemination system for PMA data by coding variables consistently across countries and survey years to facilitate pooling, trend analysis, and comparative research. So this study used the data collected in the 2018 nutrition survey of PMA, and harmonized by (IPUMS-PMA) [34]. We accessed the data through the website of IPUM-PMA (https://pma.ipums.org/pma/index.shtml) after submitting brief description about the aim of the study.

### Population and sampling procedure

PMA nutrition survey in 2018 (Burkina Faso and Kenya) used multi-stage stratified cluster sampling, where households were selected in sampled clusters, or enumeration areas (EA).

In Burkina Faso 83 enumeration areas (EAs) were sampled, and in each EA, 43 households were randomly selected. 45% of households were then randomly sub-selected. The female-child questionnaire was administered to all women age 10–49 in sub-selected households [35].

Whereas in Kenya a sample of 151 EAs were drawn first, and then in each EA, 56 households were randomly selected. 25% of households were then randomly sub-selected. Finally the female-child questionnaire was administered to all women age 10–49 years in sub-selected households [36].

This study included a sample of 3759 women of reproductive age group (age 15–49) (1868 from Burkina Faso and 1891 from Kenya).

### Data collection

The PMA2020 Burkina Faso and Kenya used innovative mobile technology to collect nationally representative nutrition data. Data was collected on women 10–49 years of age. Open Data Kit Collect (ODK), an open-source software that facilitates mobile assisted data collection, was used to create the survey platform. Local data collectors (trained in mobile assisted data collection), conducted interviews in households using smartphones equipped with ODK software. Data was then uploaded to a central server where it was validated and aggregated [37]. Data collection was conducted between June and August 2018 in Burkina Faso and between May and August 2018 in Kenya.

### Study variable measurements

#### Dependent variable

SSBs consumption which is measured by asking a woman if she drank yesterday during the day or night, whether at home or anywhere else “Any sugar-sweetened beverages like sweet fruit drinks, soft drink/fizzy drinks, sweet tea, sugar-sweetened milk tea” with an optional answer of Yes, No, or No response [38, 39].

#### Independent variables

The independent variables were chosen in the light of the current state of knowledge of their association with consumption of SSBs. The explanatory variables include age, marital status, education level, employment status, pregnancy status, ever given birth, savory and fried snack consumption, achieved minimum dietary diversity, wealth, and household food insecurity [23, 40–42].

**Minimum Dietary Diversity (MDD)** was measured by combining (aggregating) food groups and sub food groups into the 10 MDD food groups. These food groups were (1) Grains, white roots and tubers, and plantains, (2) pulses (beans, peas and lentils),3) nuts and seeds,4) Diary,5) Meat, poultry and fish,6) Eggs,7) Dark green, leafy vegetables,8) Other Vitamin-A rich fruits and Vegetables,9) Other fruits, and 10) Other Vegetables. The 10 MDD groups are then summed into a score ranging from 0 to 10. Finally each woman is then coded “yes” or “no” for scoring ≥ 5 [38].

#### Household food insecurity

is measured using an 8 items (Annual food insecurity experience scale), and then categorized as “severe” if 6 or more of the statements were true, “moderate” if 4–5 were true, and “low/none” otherwise [43, 44].

### Data management and analysis

After accessing the data from IPUM-PMA website data re-coding, labeling, cross-tabulations and analysis were done using STATA Version 14. Prior to conducting any statistical analysis, the data was weighted using sampling weight (fnqweight), and strata to keep the representativeness of the survey and to get more reliable estimates. Descriptive analysis was utilized to show the respondents’ socio-demographic characteristics (frequency and percentages – Table 1) and SSBs consumption by wealth index and household food insecurity (Bar graph). Bivariable analysis was used to indicate the possible associations between the dependent and independent variables (Table 2). Given that the hierarchical structure of the PMA data, we employed mixed-effect logistic regression for the inferential statistics. The presence of community level (EA) clustering was evidenced by Interclass Correlation Coefficient (ICC) of 0.44. Variables with a p-value ≤ 0.2 at the bivariable analysis were considered for multivariable analysis. Thus, all associations in multivariable analysis whose p-value was less than 0.05 were considered statistically significant. Results are presented as Odds Ratios (ORs) with 95% confidence intervals (CIs).

### Ethics consideration

The 2018 PMA nutrition Surveys can be downloaded from the website and are free to use by researchers for further analysis. To access the data from the IPUMS PMA website, a written request was submitted to the IPUMS. Permission was granted to use the dataset for this study; this was received from the IPUMS-PMA in February 2023. The PMA ensured international ethical standards of confidentiality, anonymity and informed consent, and availability of de-identified PMA datasets. Moreover, our study is based on publicly available secondary data. So, gaining participants consent was not applicable.

## Results

### Background and household characteristics

This study analysed data of 3,759 women aged 15–49 years old in two Sub-Saharan Africa countries (Kenya, and Burkina Faso). The median age of the women was 27 years old with interquartile range of (20,35). The majority of the study participants (68.27%) were married/living with partner at the time of data collection, and only 7.93% of them attended tertiary education. Furthermore, most (72.72%) of them were unemployed, and 38.08% of the women lived in a household with sever food insecurity Table 1.

**Table 1:** Background and household characteristics of women aged 15–49 years old in Burkina Faso, and Kenya, IPUM-PMA data 2018. [ N = 3759]

	%	Frequency (N = 3,759)
**Sociodemographic characteristics**		
Age category(years)		
15–19	20.65	776
20–34	50.84	1,911
35–49	28.52	1,072
Marital status		
Never Married	25.12	944
Currently married/living with partner	68.27	2,566
No longer living together	6.61	248
Highest level of school attended, general		
Never attended	34.93	1,313
Primary/Middle school	30.37	1,142
Secondary/post-primary	26.76	1,006
Tertiary/post-secondary	7.93	298
Employment status		
Not employed	72.72	2,734
Employed	27.28	1,025
Ever given birth		
No	27.12	1,019
Yes	72.88	2,740
Pregnancy status		
No	92.62	3,481
Yes	7.38	278
**Dietary Characteristics**		
Woman consumed yesterday: sugar-sweetened beverages		
No	49.62	1,865
Yes	50.38	1,894
Woman consumed yesterday: savory and fried snacks		
No	87.94	3,306
Yes	12.06	453
Achieved minimum dietary diversity		
No	57.52	2,162
Yes	42.48	1,597
**Household characteristics**		
Wealth score tertile		
Lowest tertile	29.8	1,120
Middle tertile	35.46	1,333
Highest tertile	34.74	1,306
Household food insecurity		
No/low	41.13	1,546
Moderate	20.79	781
Sever	38.08	1,431

### Magnitude of women’s sugar-sweetened beverages consumption

**Fig. 1:**
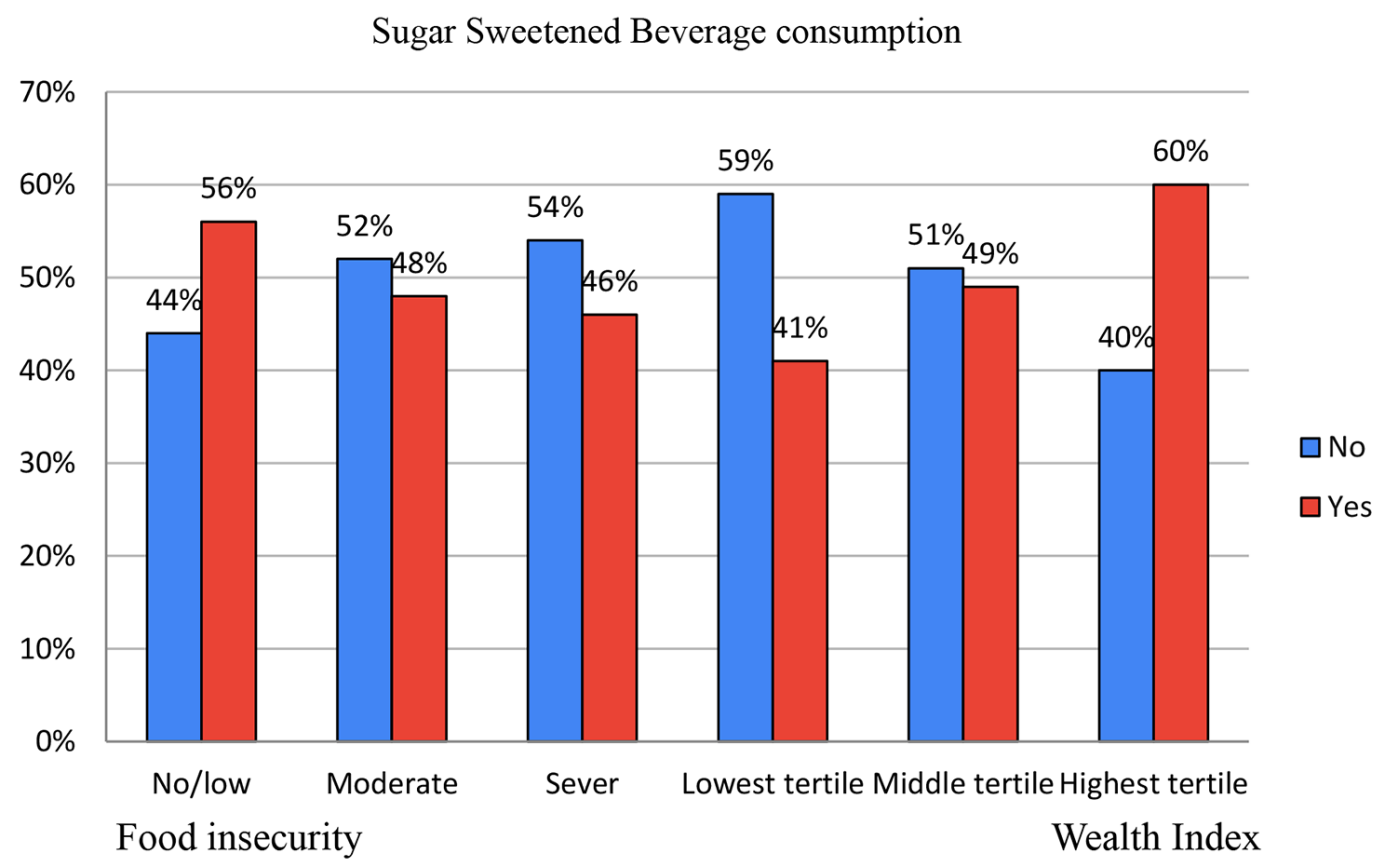
Distribution of Women’s consumption of SSBs by Food Security and Wealth Index among women aged 15–49 years old in Burkina Faso, and Kenya, IPUM-PMA data 2018. [N = 3759]

Half (50.38%) [95% CI; 46.04%, 54.71%] of women consumed SSBs in the 24-hour period of the survey date. The magnitude of SSBs consumption among women of reproductive age in two SSA countries exhibited an upward trend in correspondence with rising household income. Specifically, within the lowest wealth tertile, 41% [33%, 49%] of women reported SSB consumption, while this proportion increased to 49% [43%, 55%] within the middle wealth tertile, and further escalated to 60% [56%, 65%] within the highest wealth tertile (Fig. 1).

Additionally, SSBs consumption displayed an inverse relationship with household food insecurity levels. In households experiencing severe food insecurity, 46% [40%, 52%] of women reported SSB consumption. Within moderately food-insecure households, this figure rose to 48% [41%, 54%], and among households with no or low food insecurity, the proportion of SSB consumption was highest at 56% [50%, 61%] (Fig. 1).

### Bivariate analysis results factors associated with SSBs consumption

Bivariable mixed effect logistic regression analysis was fitted to identify variables for multivariable mixed effect logistic regression. Accordingly: Age, education level, employment status, savory and fried snack consumption, achieved minimum dietary diversity, wealth, and household food insecurity were candidate for the multivariable analysis at *p*-value < 0.2 Table 2.

**Table 2:** Bivariable mixed-effect logistic regression analysis of factors associated with women’s SSBs consumption among women aged 15–49 years old in Burkina Faso, and Kenya, IPUM-PMA data 2018. [ N = 3759]

Variables				Bivariate analysis		
				COR		CI
**Sociodemographic variables**						
Age category						
15–19				1		
20–34				1.15*		0.94–1.42
35–49				1.09		0.87–1.38
Marital status						
Never Married				1		
Currently married/living with partner				1.08		0.90–1.29
No longer living together				1.19		0.85–1.69
Highest level of school attended, general						
Never attended				1		
Primary/Middle school				1.30*		1.02–1.66
Secondary/post-primary				1.52*		1.19–1.93
Tertiary/post-secondary				1.93*		1.33–2.81
Employment status						
Not employed				1		
Employed				1.39*		1.16–1.67
Ever given birth						
No				1		
Yes				1.00		0.84–1.19
Pregnancy status						
No				1		
Yes				1.14		0.84–1.56
**Dietary Variables**						
Woman consumed yesterday: savory and fried snacks						
No				1		
Yes				1.78*		1.37–2.30
Achieved minimum dietary diversity						
No				1		
Yes				1.85*		1.54–2.22
**Household variables**						
Wealth score tertile						
Lowest tertile				1		
Middle tertile				1.23*		0.95–1.60
Highest tertile				1.93*		1.42–2.63
Household food insecurity						
No/low				1		
Moderate				0.68*		0.54–0.86
Sever				0.61*		0.49–0.75
COR = Crude Odds Ratio				* *p* < 0.2		

### Factors associated with women’s sugar-sweetened beverages consumption

In the multi-variable mixed-effect logistic regression analysis education level, employment status, savory and fried snack consumption, achieved minimum dietary diversity, and household food insecurity were found to be significant factors associated with women’s sugar-sweetened beverages consumption.

In this study level of education was significantly associated with SSBs consumption. Women with primary, and secondary education respectively were about 1.3 (AOR = 1.35; 95%CI: 1.05–1.74), and 1.5 (AOR = 1.46; 95%CI: 1.13–1.90) times more likely to consume SSBs compared to women with no education. Also, women who have some forms of employment were about 1.3 times more likely to have SSBs consumption than those who were unemployed (AOR = 1.28; 95%CI: 1.05–1.56).

Women who consumed savory and fried snack were about 1.6 times more likely to have SSBs consumption than those who did not consume savory and fried snack (AOR = 1.61, ;95%CI = 1.24–2.09). Moreover, women who achieved minimum dietary diversity were about 1.7 (AOR = 1.67; 95%CI: 1.38–2.01) times more likely to consume SSBs compared to women who did not achieve minimum dietary diversity.

Further the study revealed that household with moderate, and sever food insecurity decreased the likely of women’s SSBs consumption by 74% (AOR = 0.74, 95% CI: 0.58, 0.95), and 71% (AOR = 0.71, 95% CI: 0.56, 0.89) respectively compared to households with no/low food insecurity Table 3.

**Table 3:** Multi-variable mixed-effect logistic regression analysis of factors associated with women’s SSBs consumption among women aged 15–49 years old in Burkina Faso, and Kenya, IPUM-PMA data 2018. [ N = 3759]

Variables			
		AOR	95%CI
*Sociodemographic variables*			
Age category			
15–19		1	
20–34		1.18	0.95–1.47
35–49		1.17	0.91–1.51
Highest level of school attended, general			
Never attended		1	
Primary/Middle school		1.35***	1.05–1.74
Secondary/post-primary		1.46***	1.13–1.90
Tertiary/post-secondary		1.34	0.90–1.99
Employment status			
Not employed		1	
Employed		1.28***	1.05–1.56
**Dietary Variables**			
Woman consumed yesterday: savory and fried snacks			
No		1	
Yes		1.61***	1.24–2.09
Achieved minimum dietary diversity			
No		1	
Yes		1.67***	1.38–2.01
**Household variables**			
Wealth score tertile			
Lowest tertile		1	
Middle tertile		1.07	0.82–1.40
Highest tertile		1.35	0.97–1.87
Household food insecurity			
No/low		1	
Moderate		0.74***	0.58–0.95
Sever		0.71***	0.56–0.89
AOR = Adjusted Odds Ratio		*** *p* < 0.05	

## Discussion

This study found that 50.38% of women within the age range of 15 to 49 years in two SSA countries (Kenya and Burkina Faso) reported consuming SSBs in the 24-hour period preceding the PMA 2018 nutrition survey. This agreed with a study that found an increased trend of high levels of SSBs consumption in low and middle income countries [1]. The magnitude of SSBs consumption in this study is also consistent with a study in Australia that shows that the past week prevalence of pre-packaged drinks containing free sugar among adult population aged 18 + was 47.3% [24]. This is also consistence with a study conducted among low-income, overweight or obese pregnant women in western and southern Michigan, which revealed 48.2% consumption of SSBs [40].However the finding of the present study is lower than a study conducted among adult population in India that revealed 96.3% prevalence of SSBs consumption [45]. This discrepancy might be because of the difference in the study population i.e. the study in India were conducted among all adults aged 18–80 years old, whereas the present study included only women aged 15–49 year old [45]. The operationalization of SSBs consumption may also be the reason for the discrepancy i.e. while our study is based on single-day 24-hrs dietary recalls the study in India was based on asking how frequent respondents report drinking SSBs (daily, weekly, occasionally, or never) [45].

In the current study socio-economic variables like education level and employment status were found to be associated with SSBs consumption among women of reproductive age group in two SSA countries. Women with primary and secondary education were more likely to consume SSBs compared with women with no education. In contrast to the finding of this study, a study conducted among low-income, overweight or obese pregnant women in western and southern Michigan, and among adults aged 18–30 years in Australia highlighted that individual with low educational status were more likely to consume SSBs [40, 46]. This discrepancy might be explained by the difference in awareness of healthy food choice between women from high income (Michigan, Australia) and low income (Africa) countries. In addition, our study found that women who were employed were more likely to consume SSBs. This is consistence with a study conducted in India, São Paulo and South Africa that revealed positive association between high socioeconomic class and consumption of SSBs [45, 47, 48].

Dietary characteristics like consumption of savory and fried snack, and achieving MDD were also found to be associated with SSBs consumption. Consistence with a study conducted among adult population in South Africa, women who consumed savory and fried snack were more likely to take SSBs compare to those who did not consume savory and fried snacks [49]. This might be related to the likely of simultaneous engagement of an individual in various unhealthy behaviors [49, 50]. For instance if someone is a cigarette smoker he/she is more likely to be alcohol drinker [51], likewise, those who have been engaging in consumption of savory and fried snacks for a while, may have a strong desire to engage in other unhealthy dietary behavior like Consumption of SSBs.

Furthermore, our study found that women from household with moderate /severe food insecurity problem consume less SSBs compared to women from household with low/no food insecurity problem. This finding is also supported by a literature that showed that increased consumption patterns of SSBs in low and middle income countries is attributed to urbanization and economic growth [1]. Meaning those with no/low food insecurity problem might be economically sound and may be more likely to consume SSBs.

The strength of this study includes using appropriate statistical model for data that has hierarchical structure (women clustered in EAs). The study also describes SSBs consumption among reproductive age group using large sample size, representative of women of reproductive age group in two SSA countries (Kenya and Burkina Faso).

The limitation of this study could be that the analysis is on data from only two SSA countries. The 24-hour food consumption questionnaire may not measure the actual consumption of SSBs due to recall and, social desirability bias. Moreover, we have not included other potential factors like perceived overweight, a diagnosis of heart disease or depression due to the secondary nature of our data. Those limitations may impact on the interpretation of our findings in relation to the magnitude and correlates of SSBs intake and further studies in other African Countries with more comparable measurement of SSBs intake are needed to confirm these findings.

## Conclusions

On the whole, this study illustrates that SSBs consumption among women in two SSA countries is high. Having higher educational status, being employed, achieved minimum dietary diversity, and low/no household food in-security were found to be significantly associated with consumption of SSBs.

## Data Availability

The datasets used and/or analyzed during the current study is publicly available. The data was extracted from IPUMS-PMA website (10.18128/D081.V7.3) on February 22, 2023. Redistribution of IPUMS PMA data is not permitted under their terms of use.

## Abbreviations

AOR: Adjusted Odds Ratio
CI: Confidence Interval
COR: Crude Odds Ratio
IPUMS: Integrated Public Use Micro Data Series
PMA: Performance Monitoring for Action
SSBs: Sugar -Sweetened Beverages
